# Stress and Periodontal Disease: Reflections on the Integration of Stress and Lifestyle Management into Routine Periodontal Disease Prevention

**DOI:** 10.3390/dj14100630

**Published:** 2026-10-02

**Authors:** Blerina Zeza, Jorida Shahinas, Erdita Cenameri, Klodiana Spahiu, Doris Mingomataj, Valbona Disha, Evisa Isufaj

**Affiliations:** 1 Department of Periodontology, Faculty of Dentistry, Albanian University, 1001 Tirana, Albania; 2 Department of Medical and Health Sciences, Faculty of Medical Sciences, Barleti University, 1022 Tirana, Albania; j.shahinas@umb.edu.al (J.S.); erdita.cenameri@gmail.com (E.C.); 3 Independent Researcher, 1001 Tirana, Albania; klodianaspahiu@yahoo.com; 4 Department of Dentistry, Albanian University, 1001 Tirana, Albania; d.mingomataj@albanianuniversity.edu.al (D.M.); v.disha@albanianuniversity.edu.al (V.D.); 5 Faculty of Medical Sciences, Albanian University, 1001 Tirana, Albania; e.isufaj@au.edu.al

**Keywords:** periodontitis, periodontal diseases, primary prevention, secondary prevention, stress, lifestyle

## Abstract

This narrative review examines the role of psychological stress in periodontal disease and evaluates the evidence supporting stress-informed lifestyle and behavioural interventions within a proposed theoretical framework for stress reduction/regulation. The framework includes slow diaphragmatic breathing, positive psychological approaches, mindfulness and meditation, physical activity and stretching, appropriate daylight or sun exposure, and nutrition. For each domain, evidence is considered in relation to stress regulation, mechanistic relevance to periodontal health, and, where available, direct periodontal outcomes. The available evidence varies considerably across domains and is frequently indirect. The proposed framework represents a structured approach to stress management in periodontal care and requires prospective clinical testing to determine its feasibility, effectiveness, and potential impact on periodontal treatment outcomes.

## 1. Introduction

Periodontal diseases comprise a group of inflammatory conditions affecting the supporting structures of the teeth. Despite significant improvements in treatment techniques, advances in self-care oral hygiene instruments and materials, and the availability of both non-surgical and surgical treatments, periodontal diseases remain among the most prevalent oral diseases and continue to pose a substantial global health and economic burden [1]. Worldwide, mild and moderate forms of periodontitis affect a considerable proportion of the population [2], while severe forms, which are associated with complex treatment needs and a poorer prognosis, affect approximately 7.4% of the global population [3].

Periodontitis is generally preceded by gingival inflammation, which, in the case of dental biofilm-induced gingivitis, is reversible following effective plaque control [4,5]. Gingivitis is confined to the soft periodontal tissues and is most commonly associated with dental biofilm accumulation, although local factors, systemic conditions, and non-plaque-related causes may also contribute to gingival inflammation [5]. In contrast, patients diagnosed with periodontitis require long-term supportive care to maintain periodontal stability and prevent disease recurrence [6]. Therefore, prevention remains a fundamental component of periodontal care. Primary prevention aims to prevent the onset of periodontal inflammation, whereas secondary prevention seeks to arrest disease progression and recurrence and limit their consequences [7].

The importance of dental biofilm control in periodontal disease prevention is well established. Löe et al. demonstrated that cessation of oral hygiene measures resulted in the development of gingivitis, whereas plaque removal led to the resolution of inflammation [8]. However, the natural history of periodontal disease demonstrates substantial interindividual variability. In a 15-year study of individuals who performed no conventional oral hygiene measures, Löe et al. identified three patterns of disease progression: approximately 8% of individuals had rapid progression, 81% moderate, and 11% exhibited no progression of periodontal disease beyond gingivitis [8]. These findings indicate that, although dental biofilm is a primary etiological factor in periodontal diseases, the progression of periodontal destruction is not uniform and may be influenced by individual host susceptibility and modifying factors [9,10].

Periodontal diseases are multifactorial inflammatory conditions resulting from the interaction between microorganisms within the dental biofilm and the host immune-inflammatory response, together with additional risk and modifying factors. Accordingly, contemporary periodontal treatment emphasizes not only biofilm control but also the identification and management of relevant risk factors, as inadequate risk factor control may compromise periodontal stability [11,12]. Although dental biofilm is essential to disease development, additional direct and indirect risk and modifying factors also contribute to periodontal disease susceptibility [12]. These include host determinants, local predisposing factors, and environmental and lifestyle-related factors.

The concept that the internal biological environment may influence the development and regulation of disease has long been recognized, including in the work of Claude Bernard on the milieu intérieur [13]. In periodontal disease, changes in the subgingival environment are associated with differences in the composition and organization of microbial communities, with specific microbial complexes showing strong associations with clinical measures of periodontal disease [14].

The fundamental aim of periodontal therapy is to control inflammation and establish conditions that allow patients to maintain effective oral hygiene and achieve long-term periodontal stability. However, complete resolution of periodontal disease at all sites may not always be achieved, and long-term treatment outcomes may vary among patients [15,16,17,18,19,20,21]. This variability may partly reflect differences in patient susceptibility, adherence, and the presence of modifying factors that influence periodontal inflammation, healing, and long-term stability.

Among these factors, psychological stress warrants particular attention. Stress has been associated with periodontal disease and may negatively influence periodontal health and treatment outcomes [22,23,24,25,26,27]. Although stress is not considered a direct cause of periodontal disease, it may influence susceptibility to periodontal tissue destruction through biological and behavioral mechanisms.

Psychological stress activates neuroendocrine pathways, primarily the hypothalamic–pituitary–adrenal (HPA) axis and the sympathetic nervous system, resulting in the release of cortisol and catecholamines [23,28]. Persistent activation of these pathways may alter immune and inflammatory responses and influence periodontal tissue homeostasis [28]. Chronic stress may also contribute to immune dysregulation and increased production of pro-inflammatory mediators [29,30,31], while stress-related hormonal changes may adversely affect periodontal connective tissue and bone metabolism [32,33]. Increased sympathetic nervous system activity and heightened inflammatory responses have also been reported in patients with more severe periodontitis [34,35].

Importantly, stress-induced neuroendocrine activation may influence the oral microbiome within the subgingival biofilm through microbial endocrinology pathways [36,37,38]. Stress-related changes in catecholamines and cortisol may modify the local microbial environment and host immune responses, potentially contributing to microbial dysbiosis and periodontal inflammation [36,37,38,39]. Catecholamines may also facilitate bacterial iron acquisition by interacting with iron-binding proteins such as transferrin and lactoferrin, thereby promoting bacterial growth under iron-restricted conditions [40,41]. Bacterial sensing of host catecholamines has also been demonstrated in periodontal bacteria. In *Aggregatibacter actinomycetemcomitans*, the QseBC two-component signaling system is activated by catecholamines in the presence of iron and regulates genes involved in anaerobic metabolism, respiration, and iron storage [42]. These findings suggest that host stress mediators may influence bacterial adaptation within the periodontal environment, although the clinical relevance of this mechanism in human periodontitis requires further investigation. Conversely, periodontal microbial dysbiosis may interact with systemic immune and neuroimmune pathways, supporting a bidirectional relationship between the oral microbiome and the host [39,43,44]. The oral and gut microbiomes are interconnected through the oral–gut axis via mechanisms involving microbial translocation, metabolic signaling, and immune modulation [43,44,45]. Oral dysbiosis and the passage of oral microorganisms to the gastrointestinal tract may influence gut microbial composition and intestinal immune homeostasis [44,45], while gut dysbiosis may reciprocally affect systemic inflammatory responses and oral microbial ecology [45,46]. Through these interconnected neuroimmune and metabolic pathways, stress-related alterations in the oral microbiome may contribute to the association between periodontal inflammation and systemic inflammatory conditions [39,45,46].

In addition to its biological effects, psychological stress may influence behaviors relevant to periodontal health. Chronic stress has been associated with reduced adherence to oral hygiene practices and may coexist with other lifestyle-related behaviors that influence periodontal health, including smoking, alcohol consumption, dietary habits, and other health-related behaviors [47,48,49,50,51,52]. Consequently, psychological stress may represent a particularly relevant modifying factor because it has the potential to affect both biological susceptibility and behaviors that contribute to periodontal health and disease.

The EFP S3-level clinical practice guideline places risk-factor control within the first step of periodontal therapy and assigns this component a Grade A recommendation with strong consensus [15]. Smoking cessation and improved metabolic control of diabetes are addressed through specific evidence-based recommendations, while physical activity, dietary counselling, and lifestyle interventions for weight reduction are evaluated through dedicated clinical questions, although the available evidence was considered insufficient to support a recommendation for their use [15]. Stress reduction is likewise acknowledged as a measure that may contribute to the overall strategy for reducing patients’ risk factors. However, unlike other risk-factor domains, stress reduction is not addressed through a dedicated clinical question or recommendation in the EFP S3 guideline and was not evaluated as a distinct intervention domain in its underpinning systematic review. Thus, although stress reduction is acknowledged, a structured approach to its implementation in periodontal care remains undefined, providing part of the rationale for the present review.

**Figure 1:**
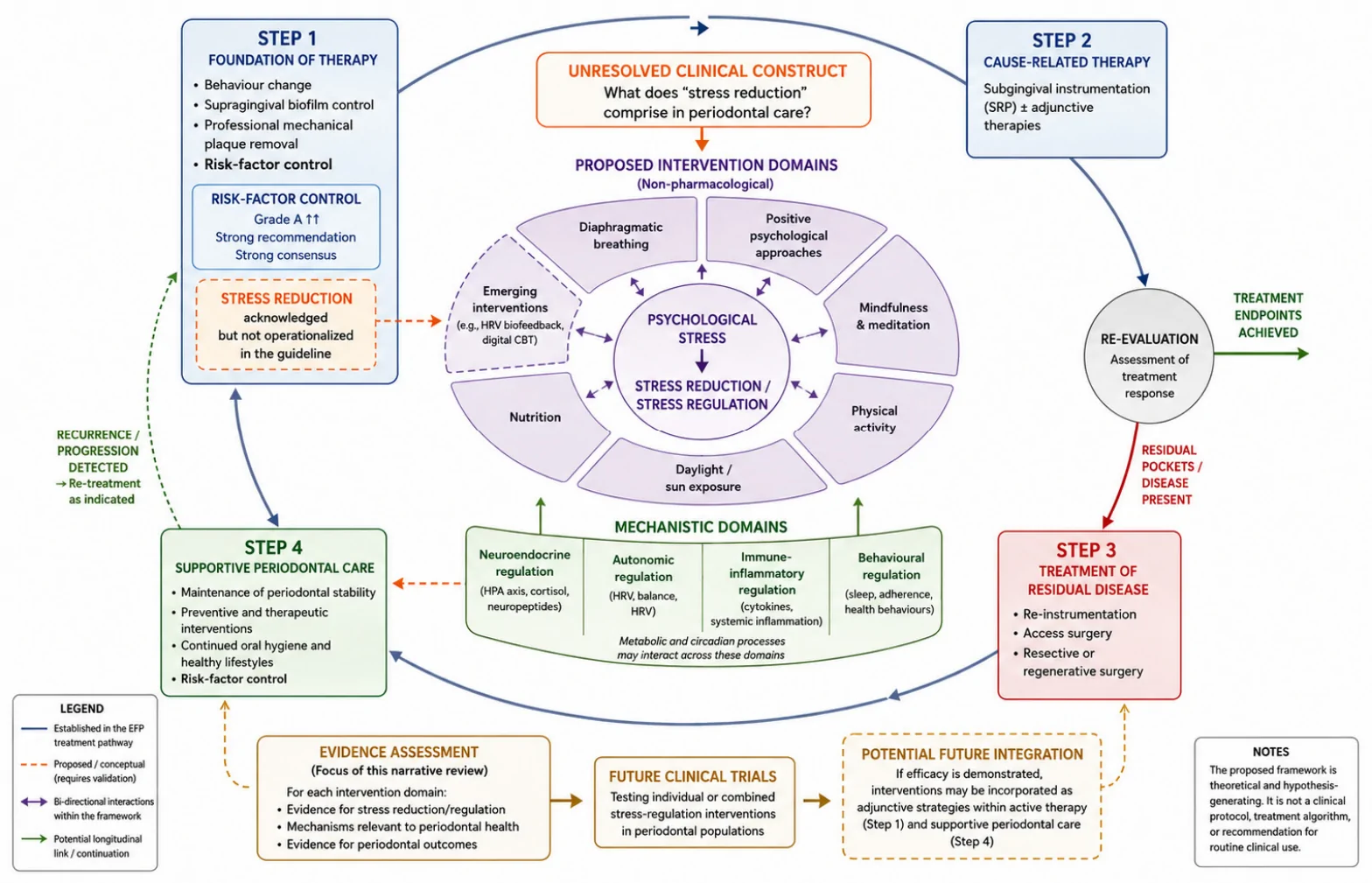
Framework of periodontal pathway treatment including EFP guidelines and the hypothetical stress management domains. Color coding distinguishes established EFP pathway components (blue), proposed stress-reduction domains (purple), mechanistic and supportive-care components (green), residual disease management (red), unresolved/conceptual elements (orange), and future evidence-generation and integration steps (gold/ochre). Arrow meanings are defined in the figure legend.

We propose a theoretical, modular framework that conceptualizes stress reduction and regulation through a set of distinct but potentially complementary non-pharmacological intervention domains, including breathing, positive mindset, mindfulness and meditation, physical activity and muscle stretching, healthy sun exposure and vitamin D status, and nutrition (Figure 1). The domains are represented in a circular, non-hierarchical configuration to reflect their potential interdependence and convergence on overlapping biological and behavioural pathways, while the modular structure allows additional interventions to be incorporated as evidence emerges.

Within this framework, the narrative review critically examines the evidence supporting each domain with respect to stress reduction or regulation, mechanistic relevance to periodontal health, and, where available, direct periodontal outcomes. The framework is intended as a theoretical and hypothesis-generating model rather than a clinical protocol or treatment algorithm. It provides a structured basis for the evaluation of stress-informed lifestyle and behavioural interventions within periodontal care and for the design of future clinical trials investigating their potential role in periodontal disease prevention and management.

## 2. Materials and Methods

A narrative approach was considered appropriate because the aim of this review was to integrate and critically interpret evidence from different disciplines and study designs to explore the relationship between psychological stress, lifestyle-related factors, and periodontal health and to develop a conceptual framework for integrated stress-informed lifestyle interventions [53,54].

This review was structured around the premise that psychological stress may influence periodontal health through both direct biological mechanisms and indirect behavioral pathways. The conceptual framework focused on six domains with potential relevance to stress regulation, general health, and periodontal disease: (a) breathing; (b) positive mindset; (c) mindfulness and meditation; (d) physical activity and muscle stretching; (e) healthy sun exposure and vitamin D status; and (f) nutrition.

The literature search was initially conducted in June 2024 and subsequently updated in 2026 through iterative searches performed throughout the development of this review. Searches were conducted in PubMed/MEDLINE, Scopus, and Web of Science. The search strategy was progressively refined as additional concepts and relevant publications emerged, and the final search update was performed on 30 June 2026. The principal search concepts included combinations of terms related to periodontal disease and psychological stress, together with terms addressing the six lifestyle domains, including “periodontitis,” “periodontal disease,” “periodontal health,” “psychological stress,” “stress,” “breathing,” “diaphragmatic breathing,” “mindfulness,” “meditation,” “positive mindset,” “positive psychology,” “physical activity,” “exercise,” “stretching,” “vitamin D,” “sunlight,” “nutrition,” “diet,” “free sugar,” “dietary habits,” “fasting,” and “intermittent fasting.” Appropriate synonyms, controlled vocabulary, and Boolean operators were adapted to the requirements of each database. Reference lists of relevant articles were also manually screened, and seminal publications were considered to identify additional studies relevant to the objectives of this review.

No publication date restrictions were applied. Priority was given to systematic reviews and meta-analyses. Primary studies, including randomized controlled trials, longitudinal studies, and well-designed observational studies, were included when they provided additional relevant evidence, complemented available systematic reviews, or addressed areas in which high-level evidence was limited. Mechanistic and experimental studies were considered when necessary to support the biological rationale underlying the relationship between psychological stress, lifestyle-related factors, and periodontal disease. Conference abstracts, editorials, opinion papers, and studies not directly relevant to the objectives of this review were excluded.

The selected literature was synthesized narratively within each of the six domains. For each domain, particular attention was given to the nature and strength of the available evidence and to whether the findings were derived directly from periodontal research or indirectly from studies in psychology, stress physiology, general health, or related disciplines. This distinction was considered particularly important because the level of periodontal-specific evidence differed substantially across the domains.

Within each domain, the evidence was critically interpreted by considering gaps in the available literature. Particular emphasis was placed on avoiding the extrapolation of indirect evidence as evidence of direct periodontal benefit. The synthesis was further informed by the authors’ clinical and academic expertise in periodontology to provide a balanced, evidence-informed overview of the potential role of stress-regulation strategies and lifestyle-related factors in periodontal prevention and management.

## 3. Results

The results are organized in six sub-sections examining lifestyle and behavioral approaches that may contribute to stress regulation and, directly or indirectly, support periodontal health.

The evidence varied substantially across the six intervention domains in terms of study design, directness of periodontal relevance, and availability of interventional data with the strongest direct periodontal evidence currently relating to nutrition. Table 1 provides an overview of the evidence identified for each domain, distinguishing the intervention proposed within the conceptual framework from the intervention or exposure actually investigated in the literature. Emphasis is placed on the source and directness of periodontal evidence, with observational or epidemiological findings considered separately from interventional studies.

### 3.1. Breathing

Breathing techniques, particularly slow and diaphragmatic breathing, have been investigated as accessible approaches for reducing physiological and psychological stress [55,56]. Controlled slow breathing may promote autonomic regulation, enhance parasympathetic activity, and attenuate sympathetic arousal. Even a single session of deep and slow breathing has been associated with improvements in vagal tone and reductions in anxiety [56]. Similarly, relaxation practices involving controlled breathing have been associated with favorable changes in cardiorespiratory dynamics and autonomic regulation [57,58,59].

The potential relevance of breathing practices to periodontal health remains less clearly established. Available periodontal studies have focused predominantly on pranayama rather than isolated diaphragmatic breathing. Pranayama typically incorporates controlled breathing within broader practices that may also include meditation, relaxation, or physical postures. Consequently, the independent contribution of diaphragmatic breathing cannot be determined from the currently available evidence.

Several intervention studies have reported improvements in periodontal clinical parameters and periodontal-related biomarkers among individuals practicing pranayama [60,61,62]. However, these studies have methodological limitations, and the available evidence remains insufficient to establish a causal effect of diaphragmatic breathing on periodontal outcomes. A recent cross-sectional study also reported more favorable periodontal health, higher salivary antioxidant capacity, and lower oxidative stress among regular pranayama practitioners compared with non-practitioners [63]. Overall, the available evidence provides preliminary support for a potential association between breathing-based practices and periodontal health but does not establish diaphragmatic breathing as an independently effective periodontal intervention.

Because diaphragmatic breathing is simple and easily incorporated into daily life, it may represent a practical strategy for stress management. However, specific breathing instructions should be presented as general stress-management guidance rather than as an evidence-based periodontal treatment.

### 3.2. Positive Mindset

Positive psychological approaches, including gratitude practices, journaling, and reflective or expressive writing, have been investigated for their potential effects on psychological well-being and stress-related outcomes [64,65,66,67,68,69,70,71]. Gratitude-based and other positive psychology interventions have been associated with improvements in measures of well-being and psychological functioning [65,66,67]. Similarly, expressive writing and journaling interventions may contribute to reductions in stress and improvements in psychological well-being, although the magnitude and consistency of these effects vary across populations and interventions [68,69,70,71].

From a clinical perspective, such approaches may encourage patients to reflect on experiences, identify positive aspects of daily life, and develop adaptive coping strategies. These interventions may therefore have potential relevance as adjunctive approaches for psychological stress management.

However, direct evidence evaluating positive mindset interventions and periodontal outcomes is currently lacking. The rationale for including this domain in an integrated stress-informed framework is therefore based primarily on its potential contribution to psychological well-being and stress regulation rather than on demonstrated direct effects on periodontal disease progression or treatment outcomes. Further research is required to determine whether interventions targeting positive psychological functioning can influence periodontal parameters.

### 3.3. Mindfulness and Meditation

Mindfulness and meditation practices have been widely investigated as approaches for reducing stress and anxiety and improving psychological well-being [72,73,74]. Their effects may involve changes in autonomic, neuroendocrine, and neurophysiological processes associated with stress regulation [73,74]. Digital applications and other technology-based approaches may also increase the accessibility of mindfulness and meditation practices [75].

Evidence specifically examining mindfulness in relation to periodontal health remains limited. A cross-sectional study reported differences in mindfulness-related measures between individuals with and without periodontitis and identified associations among perceived stress, periodontal clinical parameters, and mindfulness [24]. However, observational associations cannot establish causality. Another observational study examining mindfulness awareness, anxiety, and oral hygiene status did not provide direct evidence that greater mindfulness leads to improved periodontal health [76].

It is also important to distinguish mindfulness-specific interventions from the broader category of mind–body interventions. Much of the available periodontal literature evaluates interventions such as yoga, pranayama, or programs combining meditation with physical activity and relaxation. Therefore, improvements reported in these studies cannot be attributed specifically to mindfulness meditation.

A recent scoping review of mind–body movement-based interventions and periodontal health concluded that the available evidence is heterogeneous and limited by methodological weaknesses, precluding definitive conclusions regarding their effectiveness as adjunctive approaches to conventional periodontal therapy [77]. Overall, mindfulness and meditation may have potential relevance for stress management, but high-quality interventional studies specifically evaluating their effects on periodontal outcomes are needed.

### 3.4. Physical Activity and Muscle Stretching

Regular physical activity is associated with improvements in physical health, psychological well-being, and perceived stress [78,79]. Evidence from student and other populations suggests that regular exercise may contribute to lower stress levels and improved mental health [78,79]. Physical activity may also influence physiological stress responses, including cortisol regulation, although the effects may vary according to exercise type, intensity, duration, and individual characteristics [80,81].

Low-intensity activities such as stretching and yoga have also been investigated as approaches to stress reduction. These interventions may influence salivary stress markers, parasympathetic activity, anxiety, and perceived stress [80,81,82]. Walking and other forms of regular physical activity may additionally contribute to improvements in mental health and sleep quality [83,84].

The relevance of physical activity to periodontal health is biologically and behaviorally plausible; however, direct evidence demonstrating that physical activity or muscle stretching improves periodontal clinical outcomes remains limited. Therefore, the inclusion of this domain in the present framework is based primarily on its established benefits for stress regulation, psychological well-being, and general health, with potential indirect relevance to periodontal health. Further longitudinal and interventional studies are needed to determine whether physical activity can contribute directly to periodontal disease prevention or improve treatment outcomes.

### 3.5. Healthy Sun Exposure and Vitamin D Status

The potential relevance of healthy sun exposure to periodontal health is primarily related to its role in supporting vitamin D synthesis. Vitamin D is synthesized in the skin following exposure to ultraviolet B radiation and plays an established role in calcium and phosphorus homeostasis and skeletal health [85,86]. In addition to its classical functions, vitamin D has been investigated for potential effects on inflammatory, immune, and antimicrobial pathways [87].

Vitamin D deficiency or insufficient vitamin D status has been associated with several systemic and oral health conditions. In relation to periodontal disease, studies have investigated associations between vitamin D status, periodontitis, and periodontal treatment outcomes [88,89,90,91,92]. However, these findings should be interpreted cautiously because associations between vitamin D status and periodontal health do not demonstrate that increasing sun exposure will directly improve periodontal outcomes.

Accordingly, direct evidence supporting sun exposure itself as a periodontal intervention is currently limited. The relevance of this domain should therefore be framed primarily in terms of maintaining adequate vitamin D status rather than recommending sun exposure as a specific periodontal therapy. Vitamin D status may be influenced by several factors, including geographical location, season, skin characteristics, dietary intake, and supplementation when clinically indicated [85,86,93].

Therefore, recommendations concerning sunlight exposure should consider individual circumstances and dermatological safety. Further clinical research is required to clarify whether optimization of vitamin D status has a meaningful effect on periodontal disease progression or periodontal treatment outcomes.

### 3.6. Nutrition

Among the lifestyle domains considered in this review, nutrition currently has the strongest direct evidence in relation to periodontal health. Dietary patterns may influence periodontal inflammation through effects on the oral microbiome, host immune responses, oxidative stress, and systemic metabolic pathways [94,95].

High consumption of free sugars and refined carbohydrates may contribute to unfavorable changes in the oral environment and microbial composition [94,95]. In addition, advanced glycation end products have been implicated in inflammatory and oxidative mechanisms relevant to periodontal tissue destruction [96,97].

A systematic review examining the relationship between free-sugar intake and periodontal disease found that most of the included studies reported a higher prevalence or incidence of periodontal disease among individuals with more frequent consumption of free-sugar-containing foods or beverages [98]. However, the certainty of this evidence was limited because most included studies were observational and predominantly cross-sectional. More recently, a Mendelian randomization analysis combining NHANES and genome-wide association study data provided additional evidence supporting a potential causal relationship between higher sugar intake and periodontitis [99].

A systematic review and meta-analysis of controlled studies reported that restricting free-sugar intake was associated with a significant reduction in gingival inflammation, whereas the reduction in dental plaque did not reach statistical significance [100]. The certainty of these findings is limited by the relatively small number of studies, their sample sizes, and methodological limitations.

More recently, a randomized controlled clinical trial evaluated a four-week free-sugar avoidance intervention during periodontal therapy [101]. Both groups demonstrated clinical improvement following treatment; however, the intervention group showed a greater reduction in bleeding on probing, together with reductions in body weight, visceral fat, and free-sugar intake [101]. These findings suggest that reducing free-sugar intake may provide additional periodontal and metabolic benefits when implemented alongside conventional periodontal therapy.

Taken together, the available evidence supports an association between lower free-sugar intake and improved inflammatory periodontal outcomes. Nevertheless, the number of intervention studies remains limited, and longer-term randomized clinical trials are required to establish the magnitude, durability, and clinical relevance of these effects.

Fasting has also been investigated in relation to stress and metabolic health. A systematic review and meta-analysis suggested that fasting interventions may influence stress-related psychological outcomes, although further high-quality research is required [102]. In a recent study of healthy adults, intermittent fasting was associated with reductions in anxiety symptoms [103]. In the periodontal context, a subsequent study investigating the effects of intermittent fasting reported improvements in clinical inflammatory parameters, including reductions in bleeding on probing and the prevalence of shallow periodontal pockets; however, no significant overall changes in the composition of the subgingival microbiota were observed [104]. However, the evidence remains preliminary, and fasting cannot currently be considered an established periodontal intervention.

Experimental research has also investigated bioactive compounds such as astaxanthin in experimental periodontitis [105]. Although these findings suggest potential anti-inflammatory and bone-protective effects, evidence supporting the routine clinical use of such supplements as adjunctive periodontal therapies remains limited.

Overall, nutrition represents the lifestyle domain with the most substantial direct evidence for a relationship with periodontal inflammation and clinical periodontal outcomes. Nevertheless, further well-designed randomized controlled trials are needed to determine which specific dietary interventions are most effective and sustainable in the context of periodontal prevention and treatment.

**Table 1:** Evidence profile of the stress-informed lifestyle and behavioural intervention domains included in the proposed theoretical framework.

Domain	Proposed Intervention	Stress Reduction Evidence	Periodontal Evidence	Observational Evidence	Interventional Evidence	Critical Interpretation
**Breathing**	**Slow/diaphragmatic breathing**	Slow/deep breathing is associated with increased parasympathetic activity, reduced sympathetic arousal, improved vagal tone, and reduced anxiety/stress responses.	4 refs [60,61,62,63]; however, periodontal studies evaluate pranayama, not isolated slow diaphragmatic breathing.	1 cross-sectional study [63] reported more favourable periodontal status, higher salivary antioxidant capacity, and lower oxidative stress among regular pranayama practitioners.	3 intervention studies [60,61,62] reported improvements in periodontal clinical parameters and/or biomarkers with pranayama-based practices.	Periodontal evidence is indirect for the proposed intervention, because pranayama may incorporate meditation, relaxation, and postures. The independent periodontal effect of slow/diaphragmatic breathing remains unestablished.
**Positive Mindset**	**Gratitude practices, journaling, reflective/expressive writing, and other positive psychological exercises**	Evidence indicates improvements in psychological well-being and functioning and, in some studies, reductions in stress; effects vary across interventions and populations.	0 direct periodontal refs	None identified	None identified	Evidence currently supports stress regulation and psychological well-being, not periodontal efficacy. Periodontal relevance remains unestablished.
**Mindfulness and meditation**	**Mindfulness practice and meditation for stress regulation**	Substantial evidence supports reductions in stress and anxiety and improvements in psychological well-being, with autonomic, neuroendocrine and neurophysiological mechanisms proposed.	3 periodontal-relevant refs [24,75,77], but only limited mindfulness-specific direct evidence	2 observational studies [24,75]. Ref. [24] examined periodontitis and periodontal clinical parameters; Ref. [75] examined mindfulness, anxiety and oral-hygiene status and did not establish a periodontal effect.	No isolated mindfulness/meditation periodontal intervention identified. Intervention evidence summarized in [77] concerns broader mind–body programmes, often combining meditation with yoga, breathing or physical activity.	Stress-regulation evidence is stronger compared to mindfulness-specific periodontal evidence. Interventional studies specifically evaluating their effects on periodontal outcomes are needed
**Physical activity and muscle stretching**	**Regular physical activity, including walking/moderate activity, together with low-intensity muscle stretching**	Physical activity is associated with lower perceived stress and improved psychological well-being; stretching and low-intensity activity may influence autonomic activity, anxiety and stress biomarkers.	No directly cited periodontal primary study in the current section	None directly identified in the current evidence set	None directly identified for the proposed intervention	Strong evidence supports stress regulation and general-health effects, but direct periodontal evidence remains limited in the literature synthesized in this section.
**Daylight/sun exposure and vitamin D**	**Appropriate daylight/sun exposure**, considered in relation to physiological regulation and maintenance of adequate vitamin D status	The current section principally supports the domain through vitamin D biology rather than direct evidence that sunlight exposure itself reduces psychological stress.	4 periodontal refs [89,90,91,92] relating primarily to vitamin D status, periodontitis and/or periodontal treatment outcomes.	Associations between vitamin D status and periodontal health are reported, although the exact design of each study [89,90,91,92] is not specified in the current Results text.	No direct sun-exposure periodontal intervention identified. Any treatment-outcome evidence concerns vitamin D status rather than sunlight exposure per se.	Vitamin D evidence and sun-exposure evidence must be distinguished. Associations with vitamin D do not establish periodontal benefit from increased sunlight exposure.
**Nutrition**	**Reduction in free sugars/refined carbohydrate exposure and selected dietary strategies, including fasting/intermittent fasting as an emerging approach**	Nutrition may influence stress-related psychological, metabolic and inflammatory processes; fasting has specifically been examined in relation to stress-related psychological outcomes.	5 principal human periodontal references [99,100,101,102,104],	Ref. [99] systematic review, based predominantly on observational studies, reported associations between higher free-sugar intake and periodontal disease. Ref. [100] Mendelian-randomization analysis provided additional evidence supporting a potential causal relationship between higher sugar intake and periodontitis.	Ref. [101] systematic review/meta-analysis of controlled dietary studies found reduced gingival inflammation following restriction of free-sugar intake. Ref. [102] RCT of free-sugar avoidance during periodontal therapy reported greater improvement in bleeding on probing. Ref. [104] provides preliminary human evidence for intermittent fasting and periodontal inflammatory outcomes.	Among the proposed domains, nutrition has the most substantial direct periodontal evidence, particularly for restriction of free sugars/refined carbohydrates. The evidence supporting fasting/intermittent fasting is stronger in relation to stress and metabolic regulation, while periodontal evidence remains preliminary.

**Note:** Reference counts indicate publications cited within each intervention domain and do not represent the total number of primary studies included within systematic reviews or meta-analyses. Direct periodontal evidence refers to studies assessing periodontal clinical or periodontal-related biological outcomes and is distinguished as observational/epidemiological or interventional. Evidence derived from stress physiology, psychology, general health, or related disciplines was considered indirect with respect to periodontal outcomes. No formal grading of evidence was undertaken.

## 4. Discussion

The present narrative review identified several domains that may warrant consideration for future integration into the EFP periodontal treatment pathway, subject to further investigation and supporting evidence. The strength of evidence varies considerably between domains. Nutrition, diaphragmatic breathing, and a positive mindset represent notable areas in which evidence supporting stress regulation is growing; however, periodontal intervention studies specifically evaluating these approaches remain limited or unavailable. Regarding nutrition, reducing free-sugar intake has relatively strong clinical support and may provide additional periodontal benefits alongside conventional periodontal therapy [101]. In contrast, evidence for intermittent fasting remains preliminary, with limited and short-term periodontal studies [104]. Its safety, nutritional adequacy, and long-term sustainability in periodontal patients, particularly those with systemic comorbidities, remain insufficiently established. Therefore, intermittent fasting should not currently be considered an established periodontal intervention, and further long-term randomized clinical trials are needed.

Evidence regarding breathing and mindfulness should also be interpreted cautiously, as periodontal studies have mainly evaluated multicomponent interventions such as pranayama, yoga, or mind–body programs. Because these approaches combine several techniques, the independent effects of diaphragmatic breathing or meditation cannot be determined, limiting the development of standardized protocols based on individual techniques.

Finally, periodontal evidence concerning vitamin D primarily relates to circulating vitamin D status rather than direct sunlight exposure. Although sunlight is an important physiological source of vitamin D, cutaneous synthesis varies substantially according to latitude, altitude, season, time of day, skin pigmentation, age, and other environmental and individual factors [106]. This variability makes it difficult to define a universally appropriate level of sun exposure specifically for vitamin D optimization. Given the dermatological risks associated with UV radiation, safe sunlight exposure should therefore be recommended rather than increased exposure solely for the purpose of improving vitamin D status [106,107]. When clinically indicated, targeted supplementation under medical supervision represents a more appropriate approach. Further intervention studies are needed to establish whether correcting vitamin D deficiency improves periodontal outcomes.

Collectively, these findings highlight the need to strengthen the evidence base across all components of the proposed framework.

The clinical relevance of this perspective supports a recommendation for future research to increasingly investigate psychological stress, oral microbiota dysbiosis, and periodontal disease not as isolated and independent phenomena, but as interconnected components of a dynamic biological system. Future studies should explore how alterations in one component may directly influence the others, potentially contributing to a disruption of the physiological balance of the organism and the progression or persistence of periodontal disease.

Evidence from systematic reviews and meta-analyses generally supports a positive association between psychological stress and periodontitis. A meta-analysis conducted by Aggarwal et al. (2022), which pooled data from 14 observational studies, reported that psychological stress was associated with a significantly increased likelihood of periodontitis (pooled OR = 1.78) [108]. However, substantial statistical heterogeneity was also observed, reflecting differences in study populations, stress assessment methods, periodontal case definitions, and adjustment for potential confounding factors. Consequently, although the direction of the association appears relatively consistent, the magnitude of the effect should be interpreted cautiously.

Other systematic reviews have similarly reported a positive association between psychological stress and periodontal disease [109,110,111]. However, considerable variability exists among studies regarding the definition and conceptualization of stress. Different psychological constructs and assessment methods have been used, including perceived stress, coping behaviours, psychometric instruments, biological biomarkers, or combinations of these approaches [109,110,111]. Salivary cortisol is among the most frequently investigated biological markers of stress. The systematic review and meta-analysis by Botelho et al. specifically examined the relationship between stress, salivary cortisol, and periodontitis [109], whereas Decker et al. evaluated stress, depression, and inflammation as collective risk factors for periodontal diseases [111]. Castro et al. investigated the association between psychological stress and periodontitis and included three studies in their qualitative synthesis; two cross-sectional studies reported positive correlations between cortisol levels and periodontal parameters, including clinical attachment level (CAL) and probing depth (PD) [111].

Recent systematic reviews have further strengthened the evidence linking psychological stress and stress-related disorders with periodontal treatment outcomes. Mauland and Neupane reported approximately 0.8 mm less clinical attachment level (CAL) gain and 1.0 mm lower probing pocket depth (PPD) reduction in patients with psychological stress, although the certainty of evidence was low for CAL and very low for PPD [112]. Similar findings were reported by Villafuerte et al. [27]. However, the available evidence does not support a fixed number of additional supportive periodontal care visits based solely on stress or depression. Instead, maintenance should be individualized according to periodontal risk, treatment response, oral hygiene, adherence, and other relevant behavioural and clinical factors. Patients experiencing persistent psychological stress may benefit from closer monitoring and appropriate stress-management support as part of a personalized periodontal care plan. Further studies are needed to determine whether psychological stress should be incorporated into periodontal risk assessment. The present review builds on this emerging evidence by considering multiple modifiable lifestyle factors within an integrated periodontal framework and by distinguishing between established risk associations and the more limited evidence supporting specific lifestyle interventions. This perspective may be particularly relevant to routine periodontal care, where identification and management of modifiable behavioral risk factors can complement conventional plaque-control and periodontal treatment strategies.

From a clinical perspective, these findings support the integration of individualized lifestyle assessment and behavioural support into periodontal care. Psychological stress can be assessed using validated instruments such as the Perceived Stress Scale (PSS), with the 10-item version (PSS-10) also showing satisfactory psychometric properties in patients with periodontitis [113,114]. Nevertheless, psychological stress, depression, and dental anxiety/fear are distinct constructs that require different assessment tools, such as the PSS, Patient Health Questionnaire-9 (PHQ-9), and Modified Dental Anxiety Scale (MDAS), respectively [113,114,115,116]. Given the heterogeneity of available instruments and the limited evidence supporting their routine use in periodontal practice, further research is needed to establish the most appropriate and clinically applicable approach for stress assessment in this setting. The differential assessment of these psychological conditions was beyond the scope of the present review, which focused specifically on psychological stress and its potential relevance to periodontal disease and stress-informed interventions. Motivational interviewing has been investigated as an adjunct to periodontal therapy, with evidence suggesting that appropriately trained dental professionals can use this patient-centered approach to support behavioural change [117,118]. Motivational interviewing is not the only communication approach that may be relevant to the management of psychological distress and dental anxiety in dental practice. The Four Habits Model provides a patient-centred communication framework based on four principles: investing in the beginning of the encounter, eliciting the patient’s perspective, demonstrating empathy, and investing in the end of the consultation [119]. In addition, brief cognitive–behavioural therapy (CBT) interventions delivered by appropriately trained dentists have demonstrated effectiveness in reducing dental anxiety. In a randomized controlled trial, both dentist-administered CBT and a Four Habits Model-based approach combined with midazolam were associated with significant reductions in dental anxiety [120]. These findings suggest that structured communication strategies and brief psychological interventions may complement motivational interviewing in addressing dental anxiety and stress-related barriers to dental care. However, formal CBT requires appropriate training and should be provided or supported by qualified mental-health professionals when the patient’s psychological needs exceed the scope of routine dental care [120,121]. Nevertheless, psychological stress and related symptoms may, in some patients, exceed the scope of periodontal care and require assessment or management by appropriately qualified healthcare professionals. When the patient’s medical or psychosocial history, or clinical presentation, indicates substantial psychological distress or stress-related symptoms, the dental team should consider appropriate referral for further assessment by a physician, psychologist, or other qualified healthcare professional, as clinically indicated [15]. The role of dentists and dental hygienists may appropriately include identifying modifiable risk factors, providing brief individualized counselling, reinforcing behavioural goals, and monitoring progress, while more complex behavioural, nutritional, psychological, or substance-use problems may require multidisciplinary referral.

Professional guidance should be considered a component of comprehensive, patient-centered care rather than an additional lifestyle intervention or a substitute for conventional periodontal treatment. Its potential role is to support the identification and appropriate management of psychological or behavioral factors that may affect general health, self-care behaviors, treatment adherence, and, potentially, periodontal outcomes.

This approach allows stress-informed lifestyle strategies to be integrated into periodontal care while recognizing the boundaries of dental practice and the need for interdisciplinary collaboration when psychological support is required.

Importantly, lifestyle behaviours may also function as coping mechanisms for psychological stress and other psychosocial challenges. Consequently, recommendations to modify smoking, dietary habits, alcohol consumption, physical activity, or other behaviours should take into account the individual context in which these behaviours occur. A patient-centred approach may therefore be more appropriate than providing generic lifestyle recommendations.

Longitudinal evidence also suggests that the potential benefits of a healthier lifestyle may extend to clinically meaningful long-term periodontal outcomes. In a 6-year study of older adults, simultaneous adherence to healthier levels of smoking, physical activity, relative weight, and dietary quality was associated with a lower risk of both periodontitis incidence or progression and tooth loss. Participants with the highest healthy-lifestyle score had approximately 58% lower odds of tooth loss than those with the least healthy lifestyle [122]. Similarly, longitudinal evidence indicates that smoking cessation is associated with a progressive reduction in tooth-loss risk, with former smokers showing a substantially more favourable risk profile than current smokers [123]. Long-term periodontal-maintenance data further support a dose-dependent relationship between smoking and periodontitis-related tooth loss and suggest that the risk decreases progressively following smoking cessation [124].

Taken together, these findings suggest that lifestyle modification may have relevance beyond improvements in short-term inflammatory or clinical periodontal parameters and may contribute to the long-term preservation of the dentition. However, most available evidence remains observational, and the independent contribution of specific lifestyle interventions to tooth-loss prevention has not been established. Long-term prospective intervention studies are therefore needed to determine whether sustained stress-informed lifestyle modification can translate into measurable reductions in tooth loss and improved periodontal treatment outcomes.

Some authors advocated for the implementation of risk-based prevention strategies in periodontal clinical practice nearly two decades ago [125]. Despite the limited evidence available at that time, these authors emphasized the importance of identifying patients at increased risk and assessing stress-related factors through validated instruments evaluating perceived stress and coping strategies. Although the present review did not involve a formal systematic search or a structured evidence synthesis methodology, the body of literature considered suggests that psychological stress is a relevant factor that may influence both periodontal disease progression and periodontal treatment outcomes. Nevertheless, this interpretation should be considered in light of the heterogeneity and methodological limitations of the currently available evidence.

Clinical evaluations often focus primarily on the patient, overlooking the clinician, whose decisions, actions, communication, and overall well-being may also influence the quality of care. Dentists are exposed to considerable occupational stress, which may adversely affect their well-being, clinical judgment, and professional performance [126,127]. Maintaining a balanced lifestyle, including effective stress management, regular physical activity, and adequate rest, may support both personal well-being and professional functioning [127,128,129]. Accordingly, preventive approaches may need to extend beyond clinical procedures to include lifestyle promotion and attention to the well-being of both patients and oral healthcare professionals. The incorporation of structured education on stress management and self-care into dental curricula may also contribute to preparing future practitioners for sustainable professional practice and the delivery of high-quality patient care [128,129].

## 5. Conclusions

Overall, we can conclude that there is a need for further intervention research across each domain of the proposed framework. However, we believe that future research should not be limited to strengthening the evidence for individual interventions in isolation. This is especially important for stress management. Psychological stress is a multidimensional construct involving psychological, behavioral, physiological, and social determinants. Accordingly, interventions focusing exclusively on a single modality, such as mind–body movement practices or breathing techniques, are unlikely to capture the full complexity of stress-related mechanisms relevant to periodontal health. Given the interconnected nature of psychological stress and other modifying factors, there is a need for intervention studies evaluating integrated strategies that better reflect the complexity of behavioral and biological pathways influencing periodontal health.

## Data Availability

No new data were created or analyzed in this study. Data sharing is not applicable to this article.
